# Preventative strategies for non-contact lower limb injuries among male football players: A scoping review

**DOI:** 10.4102/sajp.v82i1.2281

**Published:** 2026-05-13

**Authors:** Siyabonga H. Kunene, Molebogeng B. Mmitsi

**Affiliations:** 1 Department of Physiotherapy, Faculty of Health Sciences, University of the Witwatersrand, Johannesburg, South Africa

**Keywords:** non-contact lower limb injuries, injury prevention strategies, male football players, exercise-based interventions, scoping review

## Abstract

**Background:**

Football is a complex team sport with high physical, tactical and technical demands, exposing players to a high risk of both contact and non-contact injuries.

**Objectives:**

To map the range of preventative strategies used to reduce non-contact lower limb injuries among male footballers.

**Method:**

Our study was conducted as a scoping review. The review was guided in five stages: (1) defining the research question; (2) identifying relevant studies; (3) selecting the topic; (4) charting and collecting data; and (5) summarising and reporting the results. The research was guided by the population, concept, context framework, focusing on professional and amateur male soccer players across various age groups. It examined a range of preventative strategies targeting non-contact lower limb injuries, recognising their critical role in reducing player exclusion because of injury. The literature search was conducted across eight electronic databases: PUBMED, CINAHL, PEDro, EBSCOhost, Cochrane Library, SPORTDiscus, Scopus and ScienceDirect.

**Results:**

Twenty-five (out of 617) studies met the inclusion criteria, and their outcomes have been presented. Outcomes revealed various injury inhibition programmes implemented for non-contact lower limb injuries among male footballers, which were: Nordic hamstring exercises, Copenhagen adduction exercise, muscle strengthening, stretching exercises, ankle taping, FIFA 11, FIFA 11+ warm-up programme, preseason preparation, running mechanics, high chronic training loads, bounding exercises and a multicomponent exercise programme.

**Conclusion:**

The study identified a range of preventative strategies commonly employed to reduce the risk of non-contact lower limb injuries among male football players.

**Clinical implications:**

Considering the varying dynamics across teams and competitive levels, these strategies can serve as a valuable framework for developing injury prevention programmes.

## Introduction

Football is a globally popular team sport characterised by complex tactical, technical and physical demands (Van Beijsterveldt et al. 2012). These demands include running, jumping, sprinting, rapid directional changes, ball kicking and physical contact with other players or objects. As a result of its intense physical and physiological nature, football is associated with a high injury rate, surpassing that of many industrial occupations (Hawkins et al. 2001).

A systematic review and meta-analysis by López-Valenciano et al. (2020) reported an overall injury incidence of 8.1 injuries per 1000 h of exposure among footballers. Match play posed a significantly higher risk (36/1000 h) compared to training (3.7/1000 h). Lower limb injuries were the most prevalent (6.8/1000 h), with muscle or tendon injuries being the most common type, often resulting from traumatic incidents rather than overuse.

Professional footballers experience high physical loads throughout their careers, placing them at elevated risk for both contact and non-contact injuries. These injuries can negatively impact their performance and career longevity. Brukner and Khan (eds. 2021) define contact injuries as those resulting from direct physical interaction with another player, object or the playing environment during training or matches. In contrast, non-contact injuries occur without direct physical contact and are typically caused by internal forces generated during movement, often influenced by biomechanical factors, fatigue or poor technique.

Various intrinsic and extrinsic factors contribute to injury risk in football. Intrinsic factors include previous injury history, age, poor flexibility, reduced muscle strength and muscle imbalances (Hägglund et al. 2013). Extrinsic factors encompass fatigue, match type (friendly versus official), location (home versus away) and outcomes, all of which may influence injury rates (Guan et al. 2021).

Larruskain et al. (2018) compared the epidemiology of injuries between elite male and female football players. Injuries acquired and individual exposure time in the male and female teams, both teams playing in the Spanish first division, were recorded by the club’s medical staff for five seasons from 2010 to 2015. The study found that the hours that men were exposed to were 20% higher compared to women. It was also discovered that training and match injury incidence were 30% – 40% higher for men than for women. Various factors between males and females were identified in addition to biological differences, which are training load and content, fixture congestion, staffing and dedication level.

Although contact injuries are more frequently reported, non-contact or overuse injuries remain a significant concern. In the systematic review and meta-analysis, Hall et al. (2020) reported that 443 of these football injuries occur across England, Spain, Uruguay and Brazil. Non-contact injuries accounted for 58.5% of cases, with 44.2% resolving within 8–28 days. Most injuries (75.4%) affected the lower limbs, with muscle/tendon tissue being the most commonly injured (29.6%). Between 31% and 41% of all non-contact injuries involved muscles, predominantly in the upper leg. Hamstring injuries represented up to 37% of all muscle injuries, followed by adductor (23%) and quadriceps femoris (19%) injuries.

The findings by Hall et al. (2020) align with clinical observations in football settings, where non-contact lower limb injuries, particularly those affecting upper leg muscles, are frequently treated. Reduction of injury incidence is crucial for enhancing team performance and minimising lost playing time (Perez-Gomez et al. 2022). Several protocols have been developed to mitigate injury risks and reduce medical and rehabilitation costs. Designing effective injury prevention programmes requires addressing the underlying risk factors. Preventative exercises have emerged as a key strategy for reducing non-contact injuries (McCall et al. 2015), with preseason conditioning training that plays an important role in injury prevention for the upcoming competitive season (Eliakim et al. 2018).

The injury prevention process involves four stages: (1) describing and identifying the extent of the injury, (2) determining contributing factors and mechanisms, (3) implementing preventative strategies and (4) returning to stage one for reassessment. While many studies have focused on the first two stages (Hawkins et al. 2001), few have explored preventative strategies. This scoping review focused on the third point of implementing preventative strategies for non-contact lower limb injuries.

When searching the literature, no scoping review has been found to map the range of preventative strategies targeting non-contact lower limb injuries in football. Our study, therefore, aimed to fill that gap by systematically reviewing the literature on strategies implemented to minimise risk factors associated with non-contact lower limb injuries among male footballers.

## Research methods and design

Prior to initiating this scoping review, the review title was registered on the Open Science Framework.

### Study type and design

Our study was conducted as a scoping review, adhering to a structured methodological approach based on the Preferred Reporting Items for Systematic reviews and Meta-Analyses (PRISMA) guidelines (Moher et al. 2009), and the framework developed by Arksey and O’Malley (2005). To ensure comprehensive and transparent reporting, the Preferred Reporting Items for Systematic reviews and Meta-Analyses extension for Scoping Reviews (PRISMA-ScR) checklist was employed to capture all essential elements relevant to scoping reviews.

The Arksey and O’Malley framework outlines five key stages in the scoping review process: (1) identifying the research question, (2) identifying relevant studies, (3) selecting studies, (4) charting the data and (5) collating, summarising and reporting the results.

### Review question

The research question was initially identified by the first author and refined through consultation with the second author, extensive literature searches and input from football clinical experts. The final research question was: What preventative strategies have been implemented to minimise the risks of non-contact lower limb injuries among male footballers?

### Identifying relevant studies

Relevant studies were identified using a multi-step search strategy involving electronic databases and reference list screening, as recommended by the Joanna Briggs Institute (JBI) methodology for scoping reviews (Peters et al. 2015). This process consisted of three distinct phases:

**Step One:** An initial exploratory search was conducted in May 2022 using the MEDLINE (PubMed) and CINAHL databases. Following JBI guidance, this preliminary search excluded grey literature and aimed to identify existing research and refine search terms. The first author used keyword combinations, such as ‘football’ OR ‘soccer’, ‘injury’, AND ‘risk factor’, AND ‘lower limb’, AND ‘non-contact’, AND ‘prevention’, AND ‘strategy’. Titles, abstracts and keywords of retrieved articles were screened to identify recurring terms, which informed the construction of a Boolean search phrase for the next phase.

**Step Two:** From May 2022 to June 2023, a comprehensive search was conducted across multiple databases, including CINAHL, MEDLINE (via PubMed), PEDro, Science Direct, SPORTDiscus with Full Text, CINAHL Plus with Full Text (EBSCO), Cochrane Library and SCOPUS. The search strategy incorporated both keyword combinations and MeSH terms, such as:

((((((((soccer) OR (football)) AND (injury)) AND (risk factor)) AND (lower limb)) AND (non-contact)) AND (prevention)) AND (strategy)) OR ((((“Soccer”[Mesh]) AND (“injuries” [Subheading] OR “Wounds and Injuries”[Mesh])) AND “Risk Factors”[Mesh]) AND “Lower Extremity”[Mesh])

Filters applied included: publication date within the last 10 years (2012–2022), human studies, English language, and male participants. A librarian was consulted to refine and adapt the search terms across all databases, ensuring consistency and thoroughness.

Search results were imported into EndNote software for reference management and duplicate removal. Databases such as CINAHL, MEDLINE and the Cochrane Database of Systematic Reviews were prioritised for their support of structured and reproducible Boolean search strategies. To capture newly published studies, the structured search was repeated in September 2023 by the first author.

### Study selection

Inclusion criteria were developed using the Population, Concept and Context (PCC) framework as recommended by Peters et al. (2015). This framework guided the formulation of the review’s objectives and eligibility parameters:

- Population: Studies involving professional and amateur male football players across various age groups were considered. An amateur player refers to any player who is registered to play for a club registered with the Football Association as an amateur in accordance with the Fédération Internationale de Football Association (FIFA) regulations (Law Insider).
- Concept: The review focused on preventative strategies targeting non-contact lower limb injuries, including interventions such as Nordic hamstring exercises (NHEs) and general strengthening programmes. These are critical in reducing the risk of injuries like hamstring and groin strains, which commonly exclude players from training and competition.
- Context: Studies were included if they examined football-related injuries in any geographical, cultural, or social setting, provided they involved male professional and/or amateur footballers.

The scope was not limited by location, allowing for a comprehensive understanding of injury prevention strategies across diverse contexts.

The study selection process was conducted independently by the first author and a research assistant, using the predefined inclusion criteria. Discrepancies were resolved through virtual discussions, and if consensus could not be reached, the second author acted as a third reviewer.

Titles and abstracts of studies identified during the third step of the search strategy were screened. Subsequently, both reviewers independently conducted full-text reviews, consistently applying the eligibility criteria. Articles not meeting the inclusion criteria were excluded following thorough examination.

**FIGURE 1:**
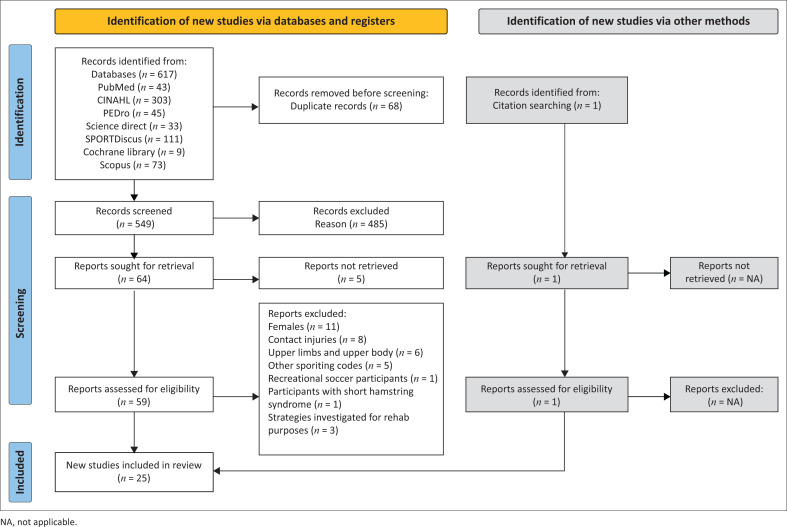
Study selection flow chart detailing the search process.

This rigorous selection process is illustrated in a flow diagram (Figure 1), structured according to PRISMA guidelines, as recommended by the JBI Reviewer’s Manual.

### Charting the data

Data extraction began in October 2023. To ensure reliability, a pilot test was conducted on three studies by the first author and reviewed by the second author. The evaluation confirmed that no modifications to the extraction method were necessary. The first author then extracted data from all eligible studies and shared the results with the research assistant for further review and discussion.

Following the JBI Reviewer’s Manual, a structured data extraction table was used. Extracted information included: author(s) and year of publication, title, design, setting, participants, findings and conclusion. These details are summarised in Table 1 in the results section.

**TABLE 1:** Summary of articles included (*N* = 25).

Author(s)	Title	Design	Setting	Participants	Findings	Conclusion
Azuma and Someya (2020)	Injury prevention effects of stretching exercise intervention by physical therapists in male high school soccer players	Randomised controlled trial	Japan	Hundred and twenty-four players from two high schools who competed in the national tournament soccer games were randomly divided into intervention and control groups	The intervention group showed significant improvements in the heel-buttock distance, straight leg raises, hip rotation and ankle dorsiflexion angles between 12 and 52 weeks	Instructed stretching exercises designed by physical therapists to address muscle tightness improved range of motion and trunk flexibility with positive effect for non-contact injuries
Bahr, Thorborg and Ekstrand (2015)	Evidence-based hamstring injury prevention is not adopted by the majority of champions league or Norwegian Premier League football teams: the Nordic Hamstring survey	Retrospective survey	Norway	Fifty professional football teams of which 32 participated in the Union of European Football Associations (UEFA) Champions League, and 18 from Tippeligaen	Of the 50 clubs, 35 reported having a formal hamstring injury prevention policy for the first squad. Education on the Nordic Hamstring programme was delivered in the 2014 season by 19 of 50 clubs	Adoption and implementation of the Nordic Hamstring exercise programme at the highest levels of male football in Europe is low, lowering the expectations on overall effect on acute hamstring injury rates
Bakken et al. (2018)	Muscle strength is a poor screening test for predicting lower extremity injuries in professional male soccer players	Case-control study	Qatar	A total of 369 professional male soccer players from 14 teams representing 42 nationalities	Greater bilateral adductor strength adjusted for body weight was associated with a lower risk for any knee injury	Strength testing cannot be recommended as a screening tool to predict injuries; however, it is possible to intervene with strength training to reduce lower extremity injuries in soccer
Best et al. (2014)	Residual mechanical effectiveness of external ankle tape before and after competitive professional soccer performance	Prospective nonrandomised test-retest design	Germany	Seventeen professional male outfield players without any signs of chronic ankle instability	Tape restricted the maximum passive inversion range of motion of the uninjured ankle significantly to 50.3%. The protection declined almost completely after 45 min	Ankle tape does not assure a protective mechanical effect against ankle sprains over the period of a soccer halftime
Cohen et al. (2015)	Angle-specific eccentric hamstring fatigue after simulated soccer	Case-control study	London	Nine male soccer players aged 25 from semi-professional soccer clubs	Hamstring strain prevention programmes for soccer players should include both exercises that specifically strengthen the hamstrings eccentrically at long lengths	Injury-risk screening could be improved by evaluating the eccentric hamstrings torque-angle profile and hamstring strength-endurance
Coratella et al. (2015)	Fatigue affects peak joint torque angle in hamstring but not in quadriceps	Case-control study	Italy	Twenty-two healthy male soccer amateur players aged between 20 and 24 years	Peak joint torque angle significantly increased in knee flexors only. No changes were found in quadriceps peak joint torque angle	Trainers and conditioners should use hamstrings lengthening exercise to adapt hamstrings length-tension profile towards a safer contraction ability
Daneshjoo et al. (2012)	The effects of injury preventive warm-up programmes on knee strength ratio in young male professional soccer players	Experimental design	Iran	Thirty-six male professional soccer players	The conventional strength ratio in the nondominant leg showed significant differences between the 11+, HarmoKnee and control group	FIFA 11+ have the potential to improve conventional strength ratio and dynamic control ratio as well as preventing knee injuries in soccer players
De Castro et al. (2013)	Incidence of decreased hip range of motion in youth soccer players and response to a stretching programme: A randomised clinical trial	Randomised clinical trial	Brazil	Two hundred and sixteen athletes of the youth professional soccer teams.	Hip-rotation range of motion decreases over the years in soccer players. Therefore, adherence to a stretching programme improved external hip-rotation in the nondominant limb	Playing soccer can restrict rotation range of motion of the hip, and adherence to stretching exercises may increase hip external range of movement and therefore decreasing the harmful effects on the hip joints
Forbes et al. (2013)	The effect of prophylactic ankle support during simulated soccer activity	Crossover study design	Germany	Ten male amateur soccer players were recruited	The application of prophylactic support significantly decreased active range of motion in plantarflexion and inversion, with tape performing better than the brace. However, tape lost its restrictive benefits within 15 minutes.	The clinical usefulness of the ankle joint prophylactic support is limited if the aim is to restrict range of motion (ROM) and improve proprioceptive capability under soccer specific conditions
Grooms et al. (2013)	Soccer-specific warm-up and lower extremity injury rates in collegiate male soccer players	Cohort study	US	Forty-one male collegiate athletes aged 18–25 years	The intervention season had reductions in the relative risk of lower extremity injury of 72%	The FIFA Medical Assessment and Research Centre (F-MARC) 11+ programme reduced overall risk and severity of lower extremity injury compared with controls in collegiate-aged male soccer athletes
Haroy et al. (2017)	Including the Copenhagen adduction exercise in the FIFA 11+ provides missing eccentric hip adduction strength effect in male soccer players	Randomised controlled trial	Norway	Forty-five eligible players from two under 19 elite male soccer teams were recruited	There was an increase in eccentric hip adduction strength in the group performing the Copenhagen adduction exercises	Including the Copenhagen adduction exercise in the FIFA 11+ programme increased eccentric hip adduction strength, and therefore potentially increasing the preventative effect on groin injuries
Ishøi et al. (2016)	Large eccentric strength increase using the Copenhagen adduction exercise in football: A randomised controlled trial	Randomised controlled trial	Denmark	Two under 19 sub-elite football teams which included 24 football players were randomised to either an 8-week supervised progressive training programme in addition to the usual training, or to continue training as usual	The intervention group demonstrated an increase in eccentric hip adduction, eccentric hip abduction and eccentric hip adduction: eccentric hip abduction ratio	Optimising the length-tension relationship using eccentric training seems to be relevant in the prevention of adductor injuries
Krist et al. (2013)	Preventive exercises reduced injury-related costs among adult male amateur soccer players: a cluster-randomised trial	Cluster randomised trial	Netherlands	Four hundred and seventy-nine adult male amateur soccer players aged between 18 and 40 years	There were no significant differences in the proportion of injured players and injury rate between the two groups. However, overall costs in the intervention group were lower compared to the control group	Exercises did not reduce the number of injuries significantly, but it significantly reduced injury-related costs
Malone et al. (2018)	High-speed running and sprinting as an injury risk factor in soccer: Can well-developed physical qualities reduce the risk?	Observational cohort study	Australia	Thirty-seven elite soccer players from one elite squad were involved in a one season study	Players who completed moderate high-speed running were at reduced injury risk compared to low high-speed running. Injury risk was higher for player who experienced large weekly changes in high-speed running and sprint running	Higher chronic training loads and better intermittent aerobic fitness offset lower limb injury risk associated with these running distances in elite soccer players
Mansfield et al. (2019)	A review advocating caution with major league soccer expansion and investment in more rehabilitation professionals	Commentary	US	Twenty-two teams participating in the Major League Soccer	Major League soccer has lengthened the regular season with the increase in number of teams participating in the league. This has resulted to increased injury rates in games compared to practice sessions	Current evidence suggests the implementation of a proper preseason preparation, one game per week frequency and implementation of injury prevention strategies would best promote player health and well-being
Pérez-Gómez et al. (2022)	Physical exercises for preventing injuries among adult male football players: A systematic review	Systematic review	Spain	A total of 2512 studies were identified initially but only 11 studies met the inclusion criteria	Injury prevention programmes in football have focused on strength training, proprioceptive training, multicomponent programmes and warm-up programmes, and have been found to be effective in lowering injury rate among male footballers	Football players can lower the incidence of match and training injuries by participating in dynamic warm-up programmes that include preventive exercises before games or during training sessions, and by adding strength, balance, and mobility training to the training sessions
Petersen et al. (2011)	Preventive effect of eccentric training on acute hamstring injuries in men’s soccer	Randomised controlled trial	Denmark	Fifty Danish male professional and amateur soccer teams (942 players) were allocated to an intervention group (461 players) or a control group (481 players)	There were 52 acute hamstring injuries in the control group compared with the 15 injuries in the intervention group	Additional eccentric hamstring exercise decreased the rate of overall, new and recurrent acute hamstring injuries in male professional and amateur soccer players
Raya-González et al. (2023)	The effects of training based on Nordic hamstring and sprint exercises on measures of physical fitness and hamstring injury prevention in U19 male soccer players	Experimental design	Spain	Forty-nine under 19 players were randomly assigned to a control or experimental group	There were significant differences in injury burden in favour of the experimental group compared to the control group, while injury incidence was not different between the two groups.	Training programme implemented in the study can improve sprint performance and reduce injury burden
Schuermans et al. (2017)	Deviating running kinematics and hamstring injury susceptibility in male soccer players: Cause or consequence?	Cohort study	Belgium	Thirty soccer players with a recent history of hamstring injury and 30 matched controls	Injury occurrence was associated with higher levels of anterior pelvic tilting and thoracic side bending throughout the swing phases of sprinting, whereas no kinematic differences during running were found when comparing players with a recent hamstring injury history with their matched controls	Although sprinting encompasses a relative risk of hamstring muscle failure in every athlete, running coordination demonstrated to be essential in hamstring injury prevention
Sebelien et al. (2014)	Effects of implementing Nordic hamstring exercises for semi-professional soccer players in Akersjus, Norway	Randomised controlled trial	Norway	Members of 10 adult, level 3 and level 4 semi-professional Norwegian soccer teams, aged between 18 and 39 years, were recruited for the study.	There was a significant difference in the number of injuries between the control and Nordic hamstring groups	Incorporation of Nordic hamstring exercise protocol into regular practice sessions may be effective in reducing the number of hamstring injuries among soccer players
Silvers-Granelli et al. (2017)	Does the FIFA 11+ injury prevention programme reduce the incidence of ACL injury in male soccer players?	Randomised controlled trial	US	Two-hundred and ninety-nine teams met the inclusion criteria, 65 institutions consented to participate with the male participants from the institution ranging from 18 to 25 years	A lower proportion of athletes in the intervention group experienced knee injuries compared with the control group including ACL injury	The FIFA 11+ programme has the potential to decrease the rate of ACL injury in competitive soccer players if implemented correctly
Silvers-Granelli et al. (2015)	Efficacy of the FIFA 11+ injury prevention programme in the collegiate male soccer player	Randomised controlled trial	US	Sixty-five teams were randomised into control group with 34 teams consisting of 850 players and intervention group with 31 teams consisting of 675 players	Control group reported 665 injuries and the intervention group reported 285 injuries	The FIFA 11+ significantly reduced injury rates by 46.1% and decreased time loss to injury by 28.6% in the competitive male collegiate soccer player
Van Beijsterveldt et al. (2012)	Effectiveness of an injury prevention programme for adult male amateur soccer players: a cluster randomised controlled trial	Cluster randomised controlled trial	Netherlands	Eleven teams consisting of 223 players in the intervention group and 12 teams in the control group consisting of 233 players	Injury incidences were almost equal between the two study groups: 9.6 per 1000 sports hours and 9.7 for the control group	No significant differences in the overall injury incidence and severity between the intervention and control groups
Van de Hoef et al. (2019)	Does a bounding exercise programme prevent hamstring injuries in adult male soccer players? – A cluster-Randomised Controlled Trial (RCT)	Randomised controlled trial	South Africa	Thirty-two soccer teams consisting of 400 players competing in the firs-class amateur league were recruited	Out of 400 players who were analysed, there were 57 players that sustained 65 hamstring injuries, but there were no statically significant differences in hamstring injury incidence or severity between the two groups	No evidence found for plyometric training in its current form to reduce hamstring injuries in amateur soccer players
Van der Horst et al. (2015)	The preventive effect of the Nordic hamstring exercise on hamstring injuries in amateur soccer players: an RCT	Randomised controlled trial	Netherlands	Male amateur soccer players from 40 teams were randomly allocated to an intervention (*n* = 20 teams, 292 players) or control group (*N* = 20 teams, 287 players)	The risk of hamstring injuries was reduced in the intervention group compared with the control group and it was statistically significant.	Incorporating the Nordic hamstring exercise protocol in regular amateur training significantly reduces hamstring injury incidence but does not reduce hamstring injury severity

Note: Please see full reference list of this article, Kunene, S.H. & Mmitsi M.B., 2026, ‘Preventative strategies for non-contact lower limb injuries among male football players: A scoping review’, *South African Journal of Physiotherapy* 82(1), a2281. https://doi.org/10.4102/sajp.v82i1.2281, for more information.

ACL, anterior cruciate ligament; FIFA, Fédération Internationale de Football Association; US, United States.

### Collating, summarising and reporting the results

After data charting, findings were systematically organised, synthesised and presented using flow diagram, table and narrative description. The narrative synthesis provided a comprehensive overview of each study, detailing aspects, such as study design, research setting, participant characteristics and identified injury predictors.

## Results

### Study selection

A total of 617 articles were first identified across seven databases. After removing 68 duplicates, 549 articles remained for title and abstract screening. Of these, 485 were excluded, leaving 64 articles for full-text retrieval. Five articles could not be retrieved; they were not published in English, and the authors did not have the resources to interpret them.

Fifty-nine articles underwent full-text review, along with one additional record identified through citation searching. Ultimately, 25 articles met the inclusion criteria.

### Summary of the studies

- **Study designs:** Among the 25 included articles, the study designs varied considerably. There were 11 randomised controlled trials, three cohort studies, three case-control studies and three experimental designs. The review included other studies which employed a retrospective survey, prospective non-randomised test-retest design, systematic review, crossover study design and commentary article. This diversity in designs reflects the broad methodological approaches used to investigate injury prevention strategies in football.
- **Study population:** All selected studies focused on male football participants, encompassing both amateur and professional players. There were no geographic restrictions applied during study selection, allowing for a globally representative sample. While some studies specified the exact number of participants, others reported only the number of teams involved, indicating variability in reporting standards across the literature.
- **Outcomes and methodology:** The reviewed studies primarily investigated preventative strategies aimed at reducing non-contact lower limb injuries among male footballers. Although the overarching focus was consistent, the methodologies employed to assess these strategies varied across studies. This included differences in intervention types, assessment tools and outcome measures, reflecting the multifaceted nature of injury prevention research in football.

### Study outcomes

Most studies included in our review were conducted in developed countries, predominantly the United States. Only one study originated from South Africa. Research was largely conducted in real-world settings, with cluster randomised controlled trials being the most common design. Strengthening interventions, particularly Nordic hamstring and Copenhagen exercises, were frequently studied and demonstrated significant effectiveness. The reviewed articles focused on preventative strategies for non-contact injuries affecting the hamstring, ankle, adductor muscles and anterior cruciate ligament (ACL). The outcomes were categorised by exercise type or intervention strategy. These included NHEs, Copenhagen Adduction Exercises (CAEs), general strengthening programmes, FIFA 11 and FIFA 11+ warm-up programmes, balance and mobility training, core stability, multicomponent programmes, bounding exercises, preseason preparation, running mechanics and chronic training loads. The following are the intervention categories and key findings:

#### Nordic hamstring exercises

Six studies that were included in the study investigated the effectiveness of NHEs in preventing hamstring injuries among football players. Bahr et al. (2015) found that despite its proven benefits, the adoption of NHE at elite levels such as the UEFA Champions League and Tippeligaen was low, limiting its impact on injury rates. Coratella et al. (2015) demonstrated that NHE improved peak joint torque angles in knee flexors following fatigue, supporting its role in enhancing hamstring safety through eccentric training. Petersen et al. (2011) reported a significant reduction in both new and recurrent hamstring injuries among male soccer players who engaged in eccentric strengthening. Similarly, Raya-González et al. (2023) observed a decrease in injury burden and improvements in sprint performance among young players following a training programme that included NHE and sprint exercises. Sebelien et al. (2014) found that incorporating NHE into regular practice sessions led to fewer injuries and increased strength and speed in semi-professional players. Van der Horst et al. (2015) confirmed that NHE significantly reduced injury incidence in amateur players, although it did not affect injury severity.

#### Copenhagen adduction exercise

The CAE has shown promising results in enhancing hip strength and potentially preventing groin injuries. Haroy et al. (2017) found that incorporating the CAE into the FIFA 11+ warm-up programme significantly increased eccentric hip adduction strength (EHAD) compared to the standard programme alone. Similarly, Ishøi et al. (2016) reported notable improvements in EHAD, eccentric hip abduction strength (EHAB), and the EHAD/EHAB ratio in the intervention group. These findings support the effectiveness of the CAE in optimising muscle balance and reducing the risk of adductor-related injuries in football players.

#### Muscle strengthening

Muscle strengthening has been explored as a strategy to reduce lower extremity injuries in football. Lauersen, Andersen and Andersen (2018) identified specific strength measures, such as quadriceps concentric peak torque and bilateral adductor strength, as being associated with injury risk.

However, they cautioned against using strength testing as a predictive screening tool. Instead, they emphasised the potential of targeted strength training to mitigate injury risk.

Complementing this, Cohen et al. (2015) found that eccentric hamstring torque declined significantly following fatigue, highlighting the importance of developing fatigue-resistant strength through eccentric training to enhance injury prevention. These two studies put emphasis on muscle strengthening to reduce the risk of non-contact injuries.

#### Stretching exercises

Stretching exercises have been shown to play a beneficial role in injury prevention among football players. Azuma and Someya (2020) demonstrated that stretching routines, administered by physical therapists, significantly improved flexibility measures such as heel-buttock distance, straight leg raises, hip rotation and ankle dorsiflexion in male high school soccer players. These improvements were associated with a reduction in non-contact injury rates. Similarly, De Castro et al. (2013) highlighted the link between decreased hip rotation and increased risk of ACL injuries. Their findings suggest that long-term biomechanical changes resulting from early and sustained soccer participation may be mitigated through consistent stretching, thereby reducing the likelihood of ACL trauma.

#### Ankle taping

Ankle taping has been examined for its role in injury prevention during soccer performance. Best et al. (2014) found that adhesive elastic ankle tape significantly reduced the maximum passive inversion range of motion in uninjured ankles, offering mechanical protection. However, this protective effect diminished after 45 min of play. Similarly, Forbes et al. (2013) reported that both tape and brace applications decreased the active range of motion in plantarflexion and inversion, with tape performing better than the brace in enhancing proprioception. Nonetheless, the tape’s restrictive benefits were lost within just 15 min of activity. These findings suggest that while ankle taping may offer short-term support, its effectiveness is limited during prolonged soccer play.

#### FIFA 11 programme

The FIFA 11 injury prevention programme, which consists of exercises aimed at improving stability, strength, coordination and flexibility, has shown mixed results in terms of effectiveness (Krist et al. 2013). Their results showed significant differences in injury rates between intervention and control groups but highlighted the programme’s cost-effectiveness for adult male amateur soccer players. Similarly, Van Beijsterveldt et al. (2012) reported comparable injury incidence and severity across both groups, suggesting that while the FIFA 11 programme may offer practical benefits, its impact on injury prevention outcomes remains inconclusive.

#### FIFA 11+ warm-up programme

Several studies have demonstrated the effectiveness of the FIFA 11+ warm-up programme in reducing injuries among male soccer players. Daneshjoo et al. (2012). Silvers-Granelli found that the FIFA 11+ and HarmoKnee programmes improved conventional strength and fast/slow speed ratios in the quadriceps of the non-dominant leg by 8%, indicating potential for knee injury prevention. Grooms et al. (2013) reported that the F-MARC 11+ programme significantly reduced lower extremity injury rates, relative risk and time lost among male collegiate players. Silvers-Granelli et al. (2015) showed a 46.1% reduction in injury rates and a 28.6% decrease in time lost in National Collegiate Athletic Association (NCAA) Division I and II male players. In a follow-up study, Silvers-Granelli et al. (2017) confirmed that the FIFA 11+ programme lowered ACL injury rates across various game settings, player positions and field types, reinforcing its effectiveness in competitive soccer.

#### Pre-season preparation

Mansfield et al. (2019) recommended structured preseason training and scheduling to reduce injury risk, especially in expanding leagues like Major League Soccer.

#### Running mechanics

Schuermans et al. (2017) linked sprinting kinematics (pelvic tilt, trunk movement) to hamstring injury risk, emphasising coordination.

#### Higher chronic training loads

Malone et al. (2018) found that consistent high-speed running and aerobic fitness reduced injury risk, while large weekly changes increased it.

#### Bounding exercise programme

Van de Hoef et al. (2019) found no significant effect of plyometric training on hamstring injury incidence or severity.

#### Multicomponent programmes

Pérez-Gómez et al. (2022) reviewed 2512 studies, with 11 meeting the inclusion criteria. Effective programmes included strength, proprioception, core stability, and warm-up routines.

## Discussion

This scoping review aimed at mapping the range of literature relating to preventative strategies implemented to minimise risk factors that are associated with non-contact lower limb injuries among male footballers. Twenty-five articles have been reviewed, and 12 preventative strategies emerged from the reviewed articles.

Nordic hamstring exercise is one of the intervention strategies used to minimise non-contact hamstring injuries. The NHE was formerly known as the ‘Russian hamstring exercise’ (Al Attar et al. 2017). The NHE is a partner-based, equipment-free movement performed on the pitch or in the gym, where one player resists a forward-falling motion from a kneeling position to eccentrically load the hamstring muscles.

Clarke and Buckley (2005) suggested that because most hamstring strains occur during eccentric contraction of the hamstring muscles, increased torque in an extended knee position may reduce the occurrence of hamstring strains. A group of scientists from the Oslo Sports Trauma Research Centre (OSTRC) hypothesised in their randomised controlled trial that NHEs could prevent some hamstring strains. In their intervention study, they found that this exercise produced a large increase in eccentric torque production in week 10 post-training, therefore minimising the risk of non-contact hamstring injuries.

Arnason et al. (2008) examined the impact of eccentric strength versus flexibility training on hamstring strain incidence in soccer players in Iceland and Norway, finding a 65% lower rate in teams using eccentric training. Bezuglov et al. (2020) further supported their use, noting their benefits for athletes engaged in high-speed running because of eccentric hamstring loading, and emphasised that those using traditional methods. Haroy et al. (2017) confirmed the effectiveness of NHEs both as a standalone and within warm-up routines. Bezuglov et al. (2020) further supported their use, noting their benefits for athletes engaged in high-speed running because of eccentric hamstring loading, and emphasised their simplicity, lack of equipment needs, and ease of implementation.

Our review study found that the FIFA 11 injury prevention programme is one of the strategies used to lower the risks of injuries. This programme was developed by the FIFA Medical Assessment and Research Centre to enhance lower limb strength and reduce injury rates in football through a series of 10 exercises targeting stability, strength, coordination and flexibility (Silvers-Granelli et al. 2015). Although two studies on FIFA 11 (Krist et al. 2013; Van Beijsterveldt 2012) showed no significant differences in injury outcomes between intervention and control groups, the programme was later modified to include the CAE and a dynamic warm-up, resulting in FIFA 11+ (F-MARC 11+).

Four studies that have evaluated FIFA 11+ reported positive outcomes, including improved quadriceps strength ratios, reduced injury rates and severity, and decreased ACL injury incidence among competitive male soccer players (Grooms et al. 2013; Silvers-Granelli et al. 2017). The inclusion of the CAE appears to be a key factor in the programme’s enhanced effectiveness, suggesting that even minor modifications can yield substantial improvements.

However, the lack of significant results in FIFA 11 may also be attributed to poor compliance or other undocumented barriers, highlighting the need for further research to understand implementation challenges and optimise injury prevention strategies.

FIFA 11 and FIFA 11+ programmes have been evaluated across various populations, including both sexes, recreational, amateur and semi-professional soccer players, as well as athletes in court-based sports like basketball (Longo et al. 2012). In a cluster randomised controlled trial involving 11 male basketball teams, Longo et al. found that teams using FIFA 11+ experienced lower injury rates per 1000 athlete-exposures compared to controls, demonstrating the programme’s effectiveness beyond football. Originally designed for football, FIFA 11+ has proven beneficial in reducing injury rates among elite basketball players, suggesting its adaptability across sports requiring frequent jumping, sprinting and rapid directional changes.

While these findings support broader application, further research is needed to validate its effectiveness in diverse athletic populations and performance contexts.

The CAE emerged as a standalone preventative strategy in our review, with two studies meeting the inclusion criteria. This simple, partner-based eccentric exercise requires no equipment and can be performed on the field, making it highly accessible (Haroy et al. 2017).

Weak hip adduction strength has been identified as an intrinsic risk factor for adductor muscle injuries in field-based sports, such as soccer, rugby and American football (Ryan, DeBurca & McCreesh 2014).

The CAE is suitable for high-intensity use before or after training sessions to reduce the risk of non-contact adductor injuries. Ishøi et al. (2016) found that this exercise significantly increased EHAD, EHAB strength and the EHAD/EHAB ratio. Bezuglov et al. (2020) reported that adductor injuries are the second most common lower limb injuries among male footballers, and the CAE helps optimise the length-tension relationship through eccentric loading. Perez-Gomez et al. (2022) further supported its effectiveness, noting a 41% reduction in groin injury risk with eccentric adductor strengthening, while Haroy et al. (2017) confirmed its role in enhancing hip adduction strength and injury prevention.

Adductor muscle injuries are the second most common muscle injuries in football (Ekstrand, Hägglund & Waldén 2011) and the leading cause of acute groin injuries in athletes (Serner et al. 2015).

Strengthening the adductors through eccentric exercises like the Copenhagen adduction protocol is therefore a relevant and practical intervention for reducing non-contact injury risk in football and other field sports. Clinicians working with male athletes should consider incorporating this protocol into training routines to target adductor strength and mitigate injury risk. Ryan et al. (2014) emphasised that low adductor strength is a significant risk factor for groin injuries, reinforcing the importance of targeted eccentric strengthening strategies such as the Copenhagen adduction.

The other preventative strategy found was muscle strengthening exercise. Four articles met the inclusion criteria and identified strength as another preventative strategy. Muscle strength is considered a significant cause of predisposing players to non-contact lower limb injuries (Lauersen et al. 2018). The management of sports injuries can be troublesome, time-consuming and expensive, but prevention in the form of strength training has proved to be accessible, effective and cost-effective for populations in sports (Lauersen et al. 2018). The role of muscle strength as a risk factor for non-contact lower limb injuries has been discussed by Creaby, Dickson and Clarke (2016). Poor or low isokinetic quadriceps and hamstring muscle strength have been associated with the risk of non-contact lower limb injuries, more particularly for acute muscle injuries and knee ligament injuries in team and non-team sports for both male and female athletes.

Lauersen et al. (2018) found that higher quadriceps concentric peak torque at 60° was linked to increased overuse injury risk, while greater bilateral adductor strength relative to body weight was associated with reduced knee injury risk, supporting strength training as a preventative measure for non-contact lower limb injuries in male footballers. Similarly, Cohen et al. (2015) emphasised the importance of developing hamstring fatigue resistance and long-length eccentric strength to lower injury incidence. These findings align with the targeted eccentric approaches of the CAE and NHE, which may help reduce adductor and hamstring injuries, respectively. Broader evidence from Lauersen et al. (2018) confirms the effectiveness of strength training across populations, including the elderly and athletes, while Coppack, Etherington and Wills (2011) demonstrated its success in reducing anterior knee pain in military recruits. Additionally, Waldén et al. (2015) reported a 64% reduction in ACL injuries through improved core stability and coordination, reinforcing strength training as a versatile and impactful strategy for clinicians aiming to prevent non-contact lower limb injuries in diverse athletic settings.

Stretching exercises are also widely recognised as a preventative strategy for non-contact lower limb injuries, particularly in sports, with various methods, such as passive, static, isometric, ballistic and proprioceptive neuromuscular facilitation, used to enhance flexibility (Thacker et al. 2004). Research has shown that soccer participation is linked to decreased hip-rotation motion and gradual musculoskeletal changes that negatively affect flexibility and performance (De Castro et al. 2013). Studies, including those by Azuma and Someya (2020), have found that stretching programmes designed by physical therapists can reduce injury rates among male high school soccer players, although further research is needed across broader populations.

Additionally, stretching may mitigate the long-term effects of reduced hip mobility in youth players, but the specific exercises most effective for injury prevention remain unclear. Despite its benefits, stretching can also cause temporary strength deficits, increased blood pressure and reduced running economy, highlighting the need for clinicians and athletes to balance its advantages with awareness of potential adverse effects (Thacker et al. 2004).

Ankle taping is used as a prophylactic strategy to reduce the risk and severity of non-contact ankle injuries in sports by stabilising the tibiotalar and subtalar joints and enhancing proprioception (Bahr & Bahr 1997; Beynnon et al. 2001; Handoll et al. 2001). It is commonly applied both preventatively and during recovery across all levels of athletic participation.

Studies have shown that while adhesive elastic tape can restrict ankle motion, its protective effect diminishes significantly within 15 min – 45 min of activity, suggesting the need for reapplication during halftime (Best et al. 2014; Forbes et al. 2013). However, limitations such as small sample sizes and non-real-world settings highlight the need for further research to determine the cost-effectiveness and optimal application of taping in reducing non-contact ankle injuries among male footballers. Despite these limitations, ankle taping remains a beneficial tool for enhancing mechanical stability and lowering injury risk.

Preseason preparation has been identified as one of the key strategies to reduce non-contact lower limb injuries, although only one article met the inclusion criteria on this topic. The abrupt expansion of the Major League Soccer (MLS) season led to a rise in such injuries, prompting recommendations for prolonged preseason training and limiting matches to one per week. Gabbett (2004) found that reducing preseason training loads in rugby league players lowered injury rates and improved aerobic capacity, suggesting that a combination of extended preparation and moderated training loads may benefit male soccer players. However, further research is needed to validate this approach specifically within soccer.

Sprint mechanics and chronic training loads also play a role in injury prevention. Schuermans et al. (2017) highlighted the risk of hamstring injuries during sprinting and emphasised the importance of coordination in prevention. Malone et al. (2018) found that elite soccer players with moderate to high exposure to high-speed and sprint running had lower injury risks than those with minimal exposure. These findings suggest that a combined focus on sprint technique and consistent training loads could enhance injury prevention strategies, warranting further investigation into their integration.

Efforts to prevent hamstring injuries have also explored exercise programmes like the NHE and the Bounding Exercise Programme (BEP), as reported in this scoping review. Van de Hoef et al. (2019) reported limited effectiveness of BEP because of low compliance, despite its aim to improve neuromuscular control and eccentric strength. Perez-Gomez (2022) reviewed various training programmes and found strength training, proprioceptive training, core stability and multicomponent programmes were effective, although BEP showed no significant benefits. The exclusion of a core stability study as a result of language barriers highlights the need for more accessible and diverse research.

### Limitations of this study

The scoping review included studies that implemented preventative strategies to minimise lower limb injuries irrespective of whether the study yielded significant or non-significant results statistically. There were also no studies found on preventative strategies for the quadriceps and gastrocnemius non-contact injuries.

## Conclusion

Non-contact lower limb injuries remain a significant challenge among male footballers, arising from a complex interplay of intrinsic and extrinsic factors. Our study identified a variety of preventative strategies currently used in clinical practice, including NHEs, CAEs, muscle strengthening, stretching, ankle taping, FIFA 11 and FIFA 11+, preseason preparation, running mechanics, higher chronic training loads, bounding and multicomponent exercise programmes. While some strategies demonstrated positive outcomes, others showed limited effectiveness in the reviewed studies. As a result of the diverse nature of these injuries and varying team dynamics across different levels of play, these strategies should serve as a guide rather than a definitive solution. Further research is needed to develop comprehensive injury prevention programmes and explore additional contributing factors to reduce both initial and recurrent non-contact injuries. A strength of our study was its rigorous review process, involving a research assistant and a third reviewer to resolve disagreements, along with a piloted data charting process to ensure consistency and reliability.
